# *Rgs6* is Required for Adult Maintenance of Dopaminergic Neurons in the Ventral Substantia Nigra

**DOI:** 10.1371/journal.pgen.1004863

**Published:** 2014-12-11

**Authors:** Panojot Bifsha, Jianqi Yang, Rory A. Fisher, Jacques Drouin

**Affiliations:** 1 Laboratoire de Génétique Moléculaire, Institut de Recherches Cliniques de Montréal (IRCM) Montréal, Quebec, Canada; 2 Division of Experimental Medicine, McGill University, Montréal, Quebec, Canada; 3 Department of Pharmacology, University of Iowa Carver College of Medicine, Iowa City, Iowa, United States of America; The University of North Carolina at Chapel Hill, United States of America

## Abstract

Parkinson disease (PD) is characterized by the preferential, but poorly understood, vulnerability to degeneration of midbrain dopaminergic (mDA) neurons in the ventral substantia nigra compacta (vSNc). These sensitive mDA neurons express Pitx3, a transcription factor that is critical for their survival during development. We used this dependence to identify, by flow cytometry and expression profiling, the negative regulator of G-protein signaling Rgs6 for its restricted expression in these neurons. In contrast to *Pitx3^−/−^* mDA neurons that die during fetal (vSNc) or post-natal (VTA) period, the vSNc mDA neurons of *Rgs6* ^−/−^ mutant mice begin to exhibit unilateral signs of degeneration at around 6 months of age, and by one year cell loss is observed in a fraction of mice. Unilateral cell loss is accompanied by contralateral degenerating neurons that exhibit smaller cell size, altered morphology and reduced dendritic network. The degenerating neurons have low levels of tyrosine hydroxylase (TH) and decreased nuclear Pitx3; accordingly, expression of many Pitx3 target gene products is altered, including Vmat2, Bdnf, Aldh1a1 (Adh2) and Fgf10. These low TH neurons also express markers of increased dopamine signaling, namely increased DAT and phospho-Erk1/2 expression. The late onset degeneration may reflect the protective action of Rgs6 against excessive DA signaling throughout life. Rgs6-dependent protection is thus critical for adult survival and maintenance of the vSNc mDA neurons that are most affected in PD.

## Author Summary

The locomotor deficits associated with Parkinson disease result from the death of a specific subset of dopamine neurons in the ventral part of the midbrain. The reason for the greater sensitivity to degeneration of those, relative to other, neurons is not clear. Prior work showed that the Pitx3 transcription factor is specifically expressed in these neurons where it has a survival role during development. The present work identified a cell signaling component, Rgs6, that is also restricted to the sensitive neurons in the midbrain and that exerts a protective function, particularly late in life. While the loss of Rgs6 function may predispose or contribute to Parkinson disease, its stimulation may provide a novel therapeutic avenue to treat Parkinson disease.

## Introduction

Parkinson disease (PD) is characterised by the progressive loss of midbrain dopaminergic (mDA) neurons [1]. Although the clinical manifestations of PD can be variable, the appearance of motor deficits is the hallmark of this neurodegenerative disease. Similarly, the etiology of PD appears to be multifactorial but one consistent feature of this disease is the greater sensitivity of ventral substantia nigra compacta (vSNc) mDA neurons to degenerate [2] as opposed to mDA neurons of the dorsal SNc (dSNc) and ventral tegmental area (VTA). The molecular basis for this preferential sensitivity remains poorly understood although work in animal models has been useful [3].

Vertebrate animal models based on genetic causes of PD, which constitute 10–20% of PD cases, have not been overly successful in reproducing the selective neurodegeneration patterns of the mDA system [4]. For example, mice with transgenic expression of human autosomal dominant mutants of α-synuclein (SNCA) or leucine-rich repeat kinase 2 (LRRK2) rarely produce mDA neurodegeneration [5], [6]. Murine loss-of-function mutations in autosomal recessive gene products for PTEN induced putative kinase 1 (*Pink*) and Parkinson protein 2 (*Park2*), have not been enlightening either, with the recent exception of Parkinson protein 7 (*Park7 or DJ-1*). Indeed, *DJ-1* ^−/−^ mice show progressive adult degeneration of SNc mDA neurons upon backcrossing to an appropriate genetic background [7], indicating that many factors are necessary in order to model polygenic diseases such as PD. However, it is noteworthy that rat models of *Pink1* and *DJ-1* loss-of-function showed progressive loss of mDA neurons [8]. Both early-onset and late-onset forms of PD bear a major histopathological hallmark, the presence of Lewy bodies, which are α-synuclein-rich protein inclusions that are also found in non-dopaminergic brain regions depending on the stage of disease progression. Many familial PD genes have widespread brain expression, without any preferential expression in mDA subpopulations [5]. In general, they all participate in similar inter-related cellular processes such as in mitochondrial function (PINK1, DJ-1, SNCA), the secretory pathway (PARK2, LRRK2) and the ubiquitin-proteasome degradation pathway (PINK1, SNCA, PARK2).

Rodent animal models based on genes that participate in development and survival of mDA neurons (Pitx3, Nurr1, Girk2, En1/2, Otx2, etc) have proved extremely useful, especially in defining the candidate cellular pathways underlying the respective differential vulnerability of SNc versus VTA mDA neuron subpopulations to toxin-induced neurodegeneration and in human pathology [9]–[11]. Notably, mouse mutants for the homeobox transcription factor Pitx3 (Entrez gene ID: 5309) are unique in that they display a selective and stereotypic pattern of mDA cell loss that resembles typical PD [12]–[14]. In particular, Pitx3-deficient mice exhibit developmental loss of Pitx3-positive Calbindin (Calb)-negative mDA neurons of the vSNc (Pitx3-dependent) while Pitx3-negative Calb-positive mDA neurons of dSNc and VTA (Pitx3-independent) remain essentially unaffected by Pitx3 deficiency [15]. This cell loss is associated with a loss of spontaneous movement that can be partially rescued by L-dopa treatment [16], [17]. Human PITX3 polymorphisms are associated with sporadic PD [18]. Pitx3 mediates its effects by regulating the expression of many genes (*Aldh1a1*, *DAT*, *Drd2*, *TH*, *Bdnf*) in a subset specific fashion [19]. A well-studied Pitx3 target gene in mDA neurons of the SNc is *Aldh1a1*, which is important for retinoic acid production and subsequent neuronal maturation and protection through regulation of TH expression [19]–[21]. Other Pitx3 target genes, such as the classical dopaminergic markers DAT, Vmat2, Drd2 are important for neurotransmitter identity and are subject to the cooperative action of Pitx3 with Nurr1 [22]. On the other hand, Pitx3 expression has been reported to be itself regulated by GDNF, especially during development [23]. GDNF is the only neurotrophic factor for which conditional inactivation in the adult mouse has provided strong evidence of its absolute requirement for the cell-autonomous survival of brain cathecholaminergic neurons, including mDA neurons [24]. Although, many studies described the implication of Pitx3 in post-natal maturation and developmental survival of mDA neurons, Pitx3 has not been conclusively linked to mechanisms of survival and maintenance of mDA neurons in the adult, especially as it pertains to degenerative processes. We hypothesized that Pitx3-controlled genes and pathways, in addition to their known role in development, may also be implicated in the neuroprotective pathway required to maintain the integrity of specific subset of mDA neurons throughout adulthood.

In a screen of expression profiling data comparing Pitx3-dependent and -independent FACS-purified mDA neurons of SNc and VTA, the Regulator of G-protein Signaling 6 (Rgs6) (Entrez Gene ID: 9628) was identified as a putative survival factor that is preferentially expressed in vSNc mDA neurons and whose expression is positively regulated by Pitx3. Rgs6 belongs to the R7 subfamily of Rgs and functions as a GTPase activating protein to terminate signaling downstream of ligand-bound G-protein coupled receptors (GPCR). It does so by accelerating the conversion from the active Gα-GTP bound state (dissociated from Gβγ subunit) to the inactive Gα-GDP bound state (associated to Gβγ subunit). The R7 subfamily of Rgs regulators, including Rgs6, are known to have preference for catalysis of pertussis toxin-sensitive Gi/o heterotrimeric G-proteins through recognition of their Gαi by a C-terminal Rgs protein domain [25]. Activated Gαi subunits inhibit adenylate cyclase such that cAMP production from ATP is halted and PKA/cAMP-dependent protein kinase pathways are inhibited. Conversely, the activated Gβγ subunit opens Girk channels to allow efflux of potassium ions outside the cell resulting in hyperpolarization [26]. The neuronal GPCRs previously associated with Gi/o proteins include dopamine receptors (Drd2, Drd3), acetylcholine receptors (m2, m4), GABA_B_ receptor, metabotropic glutamate receptors (mGluR2, 3, 4, 6, 7, 8). Thus in cerebellum and heart, phenotypes resulting from inactivation of *Rgs6* are consistent with over-activation of signaling downstream of GABA_B_, serotonin 5-HT_1A_, M2 acetylcholine receptors, respectively. [26]–[29].

In the present work, we identified *Rgs6* and investigated its role in the midbrain dopaminergic system. *Rgs6* is shown to be preferentially expressed in vSNc mDA neurons and its knockout in mice results in progressive loss and alterations of Pitx3-positive mDA neurons specifically within the vSNc of aged animals. This late-onset degeneration is associated with markers of increased Drd2 signaling, down-regulation of Pitx3 expression and deregulated expression of its target genes, *Aldh1a1*, *Bdnf*, *Vmat2*, *TH* and *Fgf10*. Further, the pattern of mDA degeneration observed in *Rgs6* ^−/−^ mice is a close phenocopy of *DJ-1* ^−/−^ mice suggesting that these two genes may act through similar pathways.

## Results

### A regulator of G-protein signaling restricted to vSNc neurons

**Figure 1:**
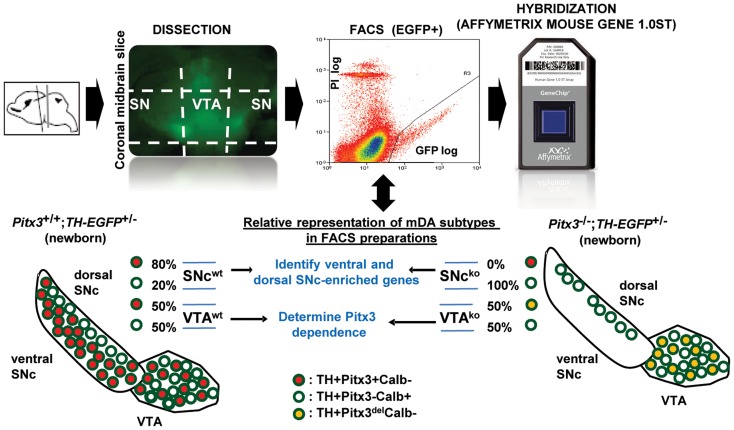
Strategy for isolation of FACS-purified Pitx3-dependent (red) and Pitx3-independent (white) mDA neurons for expression profiling analysis. Dissected SN and VTA from newborn *TH-EGFP* transgenic *Pitx3^+/+^* and *Pitx3^−/^* ^−^ mice were used for FACS purification of catecholaminergic neurons. The EGFP^+^ cells consists in various proportions (approximate % shown) of Pitx3+ (red), Pitx3− (white) and Pitx3del (yellow) mDA neurons, depending on the region dissected and mDA neuronal loss resulting from *Pitx3* inactivation. RNA from four cell preparations (SNc WT, VTA WT, SNc KO, VTA KO) were analyzed by hybridization in duplicates to Affymetrix Mouse Gene 1.0ST microarrays.

**Figure 2:**
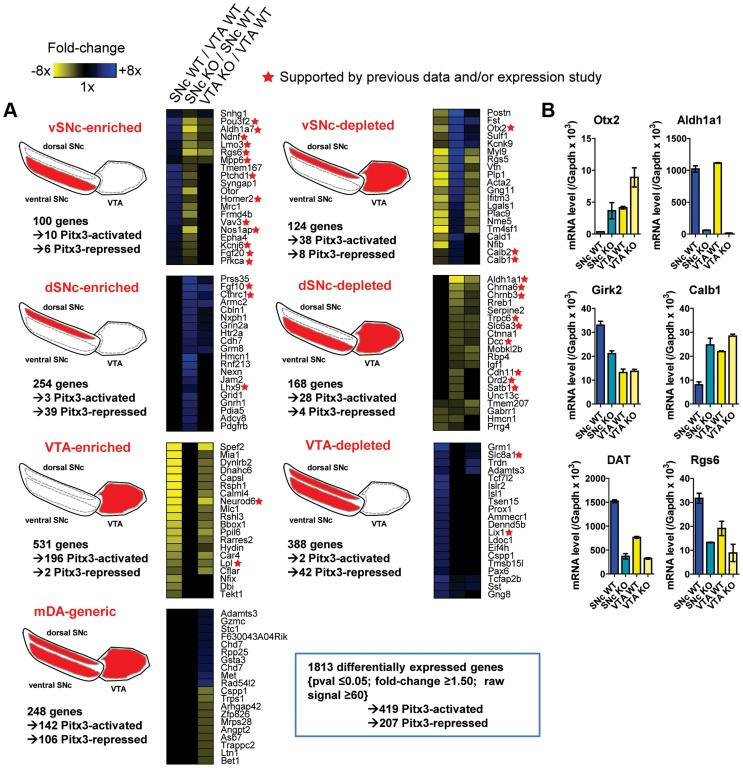
Subset-specific expression signatures of mDA neurons. (A) Differentially expressed probesets were clustered in an unbiased manner into three-dimensional Cartesian-type coordinates defined according to the relative enrichment attributes for each probeset in the comparisons between SNc WT/VTA WT, SNc KO/SNc WT and VTA KO/VTA WT. Each cluster was named according to the observed preferential expression pattern (label and shown in red in diagrams). The heatmaps show relative enrichments for 20 genes that are highly enriched and/or that have documented functions (the complete gene lists for each subset is provided in Table S1). The total number of genes (not probesets) in each cluster is indicated together with the number of those that are either activated or repressed by Pitx3 based on the VTA KO/WT comparison. (B) qRT-PCR quantification of mRNAs with preferential expression in either SN or VTA assessed in FACS-sorted cells from *WT* or *Pitx3* ^−/−^ (KO) tissues. mRNA levels are normalized relative to Gapdh mRNA. Data are shown as means ± S.D.

In order to identify genes responsible for the differential vulnerability of vSNc mDA neurons, we devised a strategy to isolate FACS-purified Pitx3-dependent and Pitx3-independent mDA neurons and compare their transcriptomes (Fig. 1). By birth, the SNc of *Pitx3* ^−/−^ pups is completely depleted of Pitx3-positive neurons but the dorsal Pitx3-negative neurons are spared [12]. After dissection of SN and VTA from midbrain slices of mice expressing TH-EGFP, FACS-sorting of TH-EGFP-positive neurons yielded a pure dSNc Pitx3-negative population from *Pitx3* ^−/−^ brains and mixed (∼80% Pitx3-positive and ∼20% Pitx3-negative) SN mDA populations from wild-type animals. The comparison of their transcriptomes defined vSNc- and dSNc-enriched genes (Fig. 2A). In VTA, Pitx3 deficiency leaves the 50% Pitx3-expressing mDA neurons intact at birth but they die within the next three months [12]. Comparison of VTA TH-EGFP cell expression profiles from *Pitx3^−/−^* and WT mice will thus identify genes which have Pitx3-dependent expression. RNA extracted from FACS-sorted cells (Fig. 1) was used to generate probes for hybridization in duplicates to Affymetrix Mouse Gene 1.0ST microarrays and determination of expression profiles. Unbiased clustering of the 1813 differentially expressed genes (fold changes >1.5, p≤0.05 and signal ≥60) into seven clusters defined genes that are expressed in specific subsets of mDA neurons and/or that are Pitx3-dependent in VTA (Fig. 2A). qRT-PCR analyses confirmed the expected enrichment for vSNc (Girk2, DAT), dSNc (Calb1) and VTA (Otx2, Calb1) markers (Fig. 2B). Further, many genes previously characterized for their subset-specific expression (marked by stars in Fig. 2A) validate the profiling data; these include Calb1/2 for dSNc, Otx2 for VTA, Kcnj6 (Girk2) for vSNc, Lpl for VTA, Aldh1a1 for vSNc, Slc6a3 (DAT) for vSNc, Lix1 for SNc [20], [30]–[32]. The complete list of genes in each cluster is provided in Table S1.

**Figure 3:**
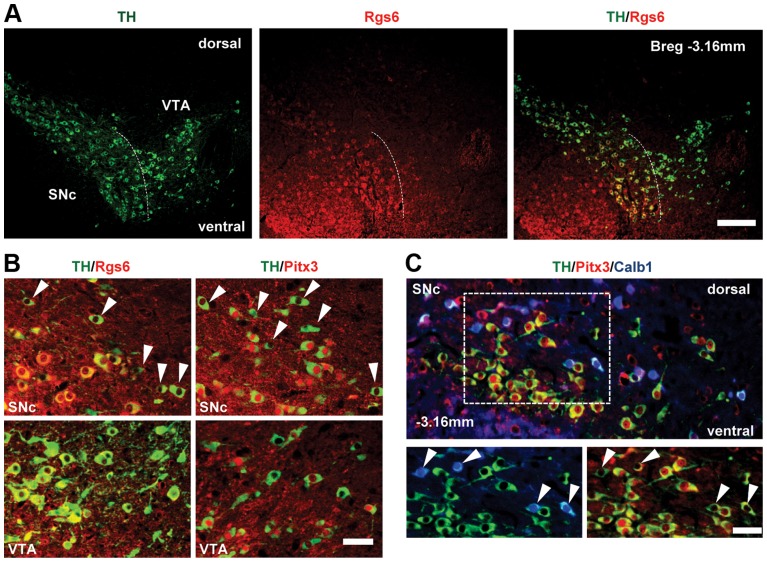
Restricted expression of Rgs6 in Pitx3-positive (Pitx3+) dopaminergic neurons of ventral SNc. (A) Rgs6 expression revealed by immunohistofluorescence (red) together with TH (green) in mDA neurons of SNc but not VTA. Scale bar 410 µm. (B) Co-immunofluorescence analysis of TH (green) and Rgs6 or Pitx3 (red, as indicated) in SNc and VTA of adult mice. Arrowheads point to Pitx3-negative or Rgs6-negative mDA neurons in dorsal SNc. Scale bar 100 µm. (C) Triple immunofluorescence staining for TH (green), Pitx3 (red) and Calb1 (blue) on tissue sections of adult mice indicates that the majority of TH+Pitx3− cells of dSNc (arrowheads) are positive for Calb1, while TH+Pitx3+ cells of vSNc are negative for Calb1, consistent with depiction in A. Scale bar 100 µm.

We chose to investigate genes enriched in vSNc and Pitx3-dependent. Rgs6 immediately appeared as an interesting candidate because it is known to negatively modulate signaling downstream of heterotrimeric Gi protein-coupled receptors through its instrinsic GTPase-stimulating protein activity [26], [28], [29]. The Rgs6 protein was detected by immunohistofluorescence in TH-positive SNc mDA neurons and not in VTA (Fig. 3A). Most dSNc mDA neurons were negative for Rgs6 (Fig. 3B upper left, arrowheads) as were VTA cells (Fig. 3B, bottom left). The SNc distribution of Rgs6 is very similar to that of Pitx3 but they differ in VTA (Fig. 3B). Triple immunohistofluorescence staining against TH, Calb1 and Pitx3 (Fig. 3C) showed that the majority of Pitx3-positive vSNc mDA neurons are negative for Calb1 whereas dSNc mDA neurons are calbindin-positive. The SNc thus has two major subsets of mDA neurons with differential vulnerability to *Pitx3* knockout: TH+/Pitx3+/Rgs6+/Calb− neurons in vSNc (Pitx3-dependent, PD vulnerable) and TH+/Pitx3−/Rgs6−/Calb+ cells in dSNc (Pitx3-independent, PD resistant).

### Neuronal degeneration in vSNc of *Rgs6* ^−/−^ mice

In order to define the *in vivo* role of Rgs6, we investigated the mDA system of *Rgs6* ^−/−^ mice by TH immunohistochemistry at 6, 180 and 356 days of age (Table S2). Only 1 y-old *Rgs6* ^−/−^ midbrains were markedly different from controls and we could identify two major phenotypes in different mice.

**Figure 4:**
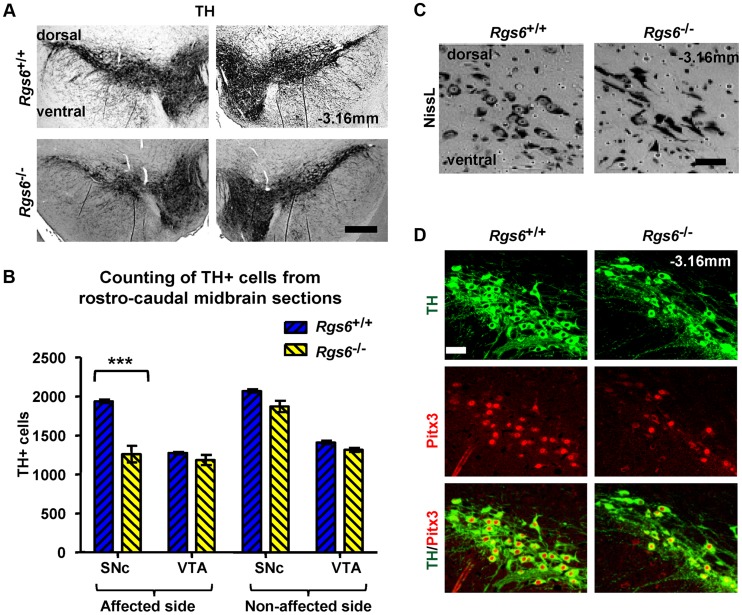
Unilateral loss of Pitx3-positive dopaminergic neurons in ventral SNc of *Rgs6* ^−/−^ mice. (A) Immunoperoxidase staining for TH on representative coronal midbrain sections showing less SNc TH+ neurons on one side of *Rgs6* ^−/−^ mice at 1 year of age compared to sib control. Sections are identified with Bregma position. Scale bar 400 µm. (B) Number of TH+ cells in SNc and VTA of TH-stained coronal sections across midbrain (every 30 µm). Cell counts are represented as means +/− S.D. (***p<0.005). (C) Nissl staining of vSNc sections contiguous to A shows fewer cell bodies and abnormal elongated neurons in *Rgs6* ^−/−^ mice compared to control. Scale bar 100 µm. (D) Double immunofluorescence staining for TH (green) and Pitx3 (red) on sections contiguous to A showing a marked loss of TH+Pitx3+ cells in *Rgs6^−/−^* mice. Scale bar 100 µm.

The first phenotype was a partial loss of SNc TH-positive cells on one side of the brain (random unilateral) (Fig. 4A and Figure S1). Quantification of TH-positive cells in SNc and VTA indicated a loss of about 35±6% (SD) in the SNc of the affected side (Fig. 4B). The loss of TH-positive cells was further supported by fewer Nissl-positive cells in SNc of *Rgs6* ^−/−^ mice (Fig. 4C). In addition, the Nissl stain revealed the presence of cells with abnormal elongated morphology that were found unilaterally within the SNc (Fig. 4C). The loss of TH-positive cells is correlated with a loss of Pitx3-positive cells (Fig. 4D).

**Figure 5:**
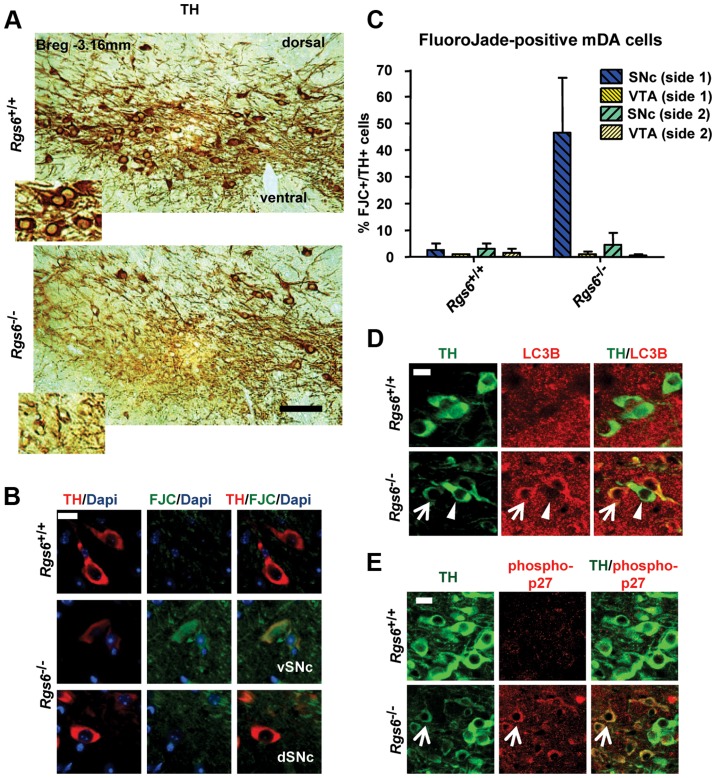
Unilateral degeneration of vSNc neurons in a subset of *Rgs6−/−* mice. (A) Immunoperoxidase staining for TH on representative coronal midbrain sections showing dysmorphic TH+ neurons (low TH staining, TH^low^, inset) in ventral SNc of *Rgs6* ^−/−^ mice. The dSNc and VTA mDA neurons are unaffected (strong/normal TH staining, inset). Scale bar 200 µm. (B) Triple staining for TH (red), Dapi (blue) and Fluoro-Jade C (FJC, green) showing presence of degenerating TH^low^ cells in vSNc of 1 y-old *Rgs6* ^−/−^ mice (middle panels) and not in control vSNc (upper panels) or in dorsal SNc (lower panels). Scale bar 20 µm. (C) Bilateral cell counts of FJC^+^ mDA neurons in SNc and VTA of coronal sections from control and 1 y-old *Rgs6* ^−/−^ mice. (D) Co-immunostaining for TH (green) and LC3B (red) in vSNc of *WT* and *Rgs6* ^−/−^ mice. Scale bar 20 µm. Arrowheads indicate unaffected neurons while arrows point to TH^low^ cells. (E) Co-immunostaining for TH (green) and phosphorylated p27^Kip1^ (phospho-p27, red) in vSNc of *WT* and *Rgs6* ^−/−^ mice. Scale bar 20 µm.

A second group of 1 y-old *Rgs6* ^−/−^ mice exhibited dysmorphic mDA neurons that displayed low levels of TH immunoreactivity (TH^low^), aberrant morphology, pronounced cell shrinkage and disrupted TH-positive fiber network (Fig. 5A and Figure S2). These dysmorphic neurons were all localized in the vSNc, while mDA neurons in dSNc and VTA had normal appearance and unaffected Calb1 expression (Figure S3).

We then determined whether the *Rgs6* ^−/−^ dysmorphic mDA neurons are undergoing degeneration by staining with Fluoro-Jade C (FCJ), as previously shown in the MPTP or 6-OHDA-induced mouse PD models [33], [34] and *zitter* mutant rats [34]. A high proportion of the dysmorphic mDA neurons stained positive for FJC (Fig. 5B, C) and are TH^low^. Cell counts indicated that degeneration is mostly unilateral and limited to the vSNc (Fig. 5C). Some 180 days-old *Rgs6−/−* mice exhibited mild unilateral degeneration in the most lateral part of SNc, while newborn mice did not, supporting the progressive appearance of degeneration with age (Table S2).

### Expression of pathological markers and familial PD gene products in *Rgs6^−/−^* vSNc neurons

**Figure 6:**
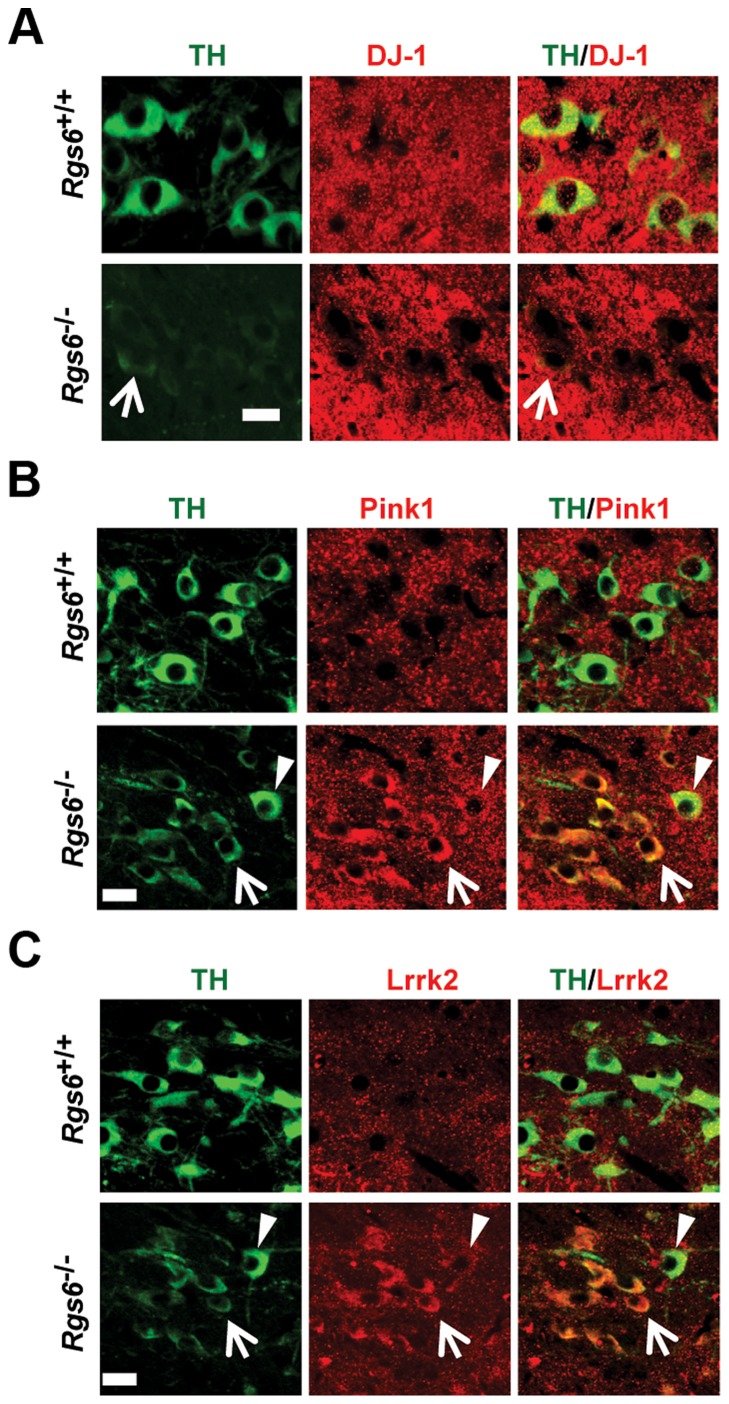
Expression of familial PD genes is altered in degenerating neurons of vSNc. Double immunofluorescence staining against TH (green) and (A) DJ-1 (red) or (B) Pink1 (red) or (C) Lrrk2 (red) in SNc of control and 1 y-old *Rgs6* ^−/−^ mice that display dysmorphic TH^low^ mDA neurons. Arrowheads indicate unaffected neurons while arrows point to TH^low^ cells. Scale bar 20 µm.

We then characterized degenerating mDA neurons in *Rgs6^−/−^* mice for the presence of pathologocial markers observed in PD and in other neurodegenerative diseases. Notably since degenerating neurons in PD suffer from oxidative stress and increased autophagocytosis due to the presence of protein aggregates, we assessed and observed increased expression of LC3B, a marker of activated autophagosome in degenerating neurons (Fig. 5D). In order to further characterize the degenerating neurons, we verified expression of key genes implicated in development of familial forms of PD [5]. For example, *DJ-1* and *PINK1* are two genes whose mutated forms cause PD in an autosomal recessive manner and *LRRK2* is the most frequently mutated gene causing PD and it acts in an autosomal-dominant fashion. *Pink1* and *Lrrk2* mouse null mutants do not however display degeneration of mDA neurons [5], [35] but a rat model of *Pink1* knockout does [8]. Surprisingly, the TH^low^ neurons of *Rgs6^−/−^* vSNc specifically exhibit decreased DJ-1 expression, while Pink1 and Lrrk2 expression is increased in those same neurons (Fig. 6); this contrasts with the fairly widespread expression of DJ-1 [36], Pink1 and Lrrk2 [5], [37] in midbrain neurons. Interestingly, the late-onset degeneration observed in *Rgs6^−/−^* midbrain appears to be a close phenocopy of the *DJ-1^−/−^* mice [7], especially in terms of the initial unilateral nature of defects in aging SNc mDA neurons and the kinetics of cell loss. The loss of DJ-1 may thus contribute to Rgs6-dependent degeneration.

Degenerating neurons were shown to overexpress the phosphorylated cell cycle inhibitor p27^Kip1^ in Alzheimer's disease [38]. Interestingly, Rgs6 was implicated in control of cell cycle and apoptosis [39] and we observed cytoplasmic accumulation of phospho-p27^Kip1^ only in degenerating TH^low^ mDA neurons of one year-old *Rgs6^−/−^* mice (Fig. 5E).

### Reduced Pitx3-dependent gene expression in *Rgs6* ^−/−^ vSNc neurons

**Figure 7:**
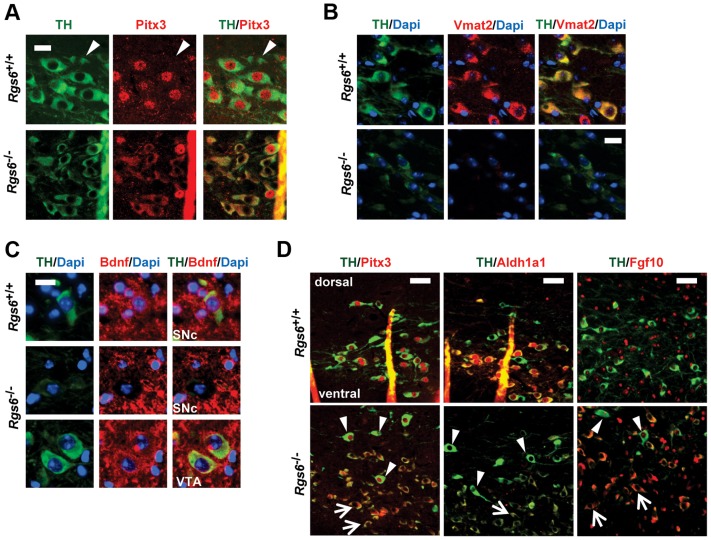
Reduced expression of Pitx3 and its target genes in degenerating neurons. (A) Double immunofluorescence staining against TH (green) and Pitx3 (red) in SNc of control and 1 y-old *Rgs6* ^−/−^ mice that display dysmorphic mDA neurons. Arrowheads indicate Pitx3-negative neurons of dSNc. Scale bar 20 µm. (B) Co-immunofluorescence staining against TH (green), nuclear Dapi (blue) and Vmat2 (red) in SNc of *WT* and *Rgs6* ^−/−^ mice. Scale bar 20 µm. (C) Co-immunofluorescence staining against TH (green), nuclear Dapi (blue) and Bdnf (red) in SNc and VTA of *WT* and *Rgs6* ^−/−^ mice. Scale bar 20 µm. (D) Double immunofluorescence staining against TH (green) and Pitx3, Aldh1a1, Fgf10 (red) in control and 1 y-old *Rgs6* ^−/−^ mice that display dysmorphic mDA neurons. Arrowheads indicate unaffected dSNc neurons and arrows point to affected vSNc neurons. Scale bar 100 µm.

In order to define molecular correlates of Rgs6-dependent degeneration, we assessed Pitx3 expression that was previously shown to have a survival function in SNc [12]. Nuclear Pitx3 was greatly diminished in the dysmorphic mDA neurons while cytoplasmic Pitx3 staining increased, suggesting a shift in sub-cellular localization observed with two different polyclonal Pitx3 antibodies in different cells of the same sections (Fig. 7A). We assessed protein levels of some Pitx3 target genes by immunohistochemistry: these include Bdnf, Aldh1a1 (Adh2), TH, Drd2, DAT, Vmat2 and Fgf10 [19], [23], [40]. The expression of Vmat2 and Bdnf is decreased in TH^low^ neurons (Fig. 7B, C). Expression of Aldh1a1 (Adh2) is also decreased in degenerating vSNc mDA neurons (Fig. 7D): this decrease may in part account for the low TH expression [19]. Our profiling data suggested that *Fgf10* expression is repressed by Pitx3 in VTA and indeed, we found de-repression of Fgf10 expression only in TH^low^ degenerating mDA neurons (Fig. 7D). At post-natal day 6 (not shown), we did not observe any change in expression of these genes consistent with a late onset phenotype.

### Markers of dopaminergic signalling in *Rgs6* ^−/−^ midbrains

**Figure 8:**
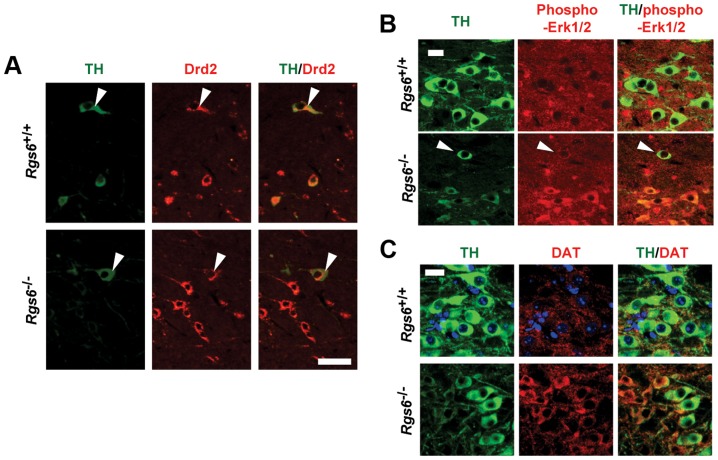
Drd2-related changes of gene expression in degenerating neurons of vSNc. (A) Immunohistofluorescence staining for TH and Drd2 in coronal sections of 1 y-old Rgs6^−/−^ mice and WT controls. mDA neurons of vSNc express higher levels of glycosylated dopamine receptor D2 (Drd2) than those of dSNc (arrowheads). Scale bar 50 µm. (B) Phospho-Erk1/2 (red) staining is only present in degenerating vSNc TH^low^ (green) neurons of *Rgs6^−/−^* mice and not in *WT* controls. Scale bar 20 µm. Arrowheads indicate unaffected neurons. (C) DAT (red) staining is stronger in degenerating vSNc TH^low^ (green) neurons of *Rgs6^−/−^* mice than in *WT* controls. Scale bar 20 µm.

**Figure 9:**
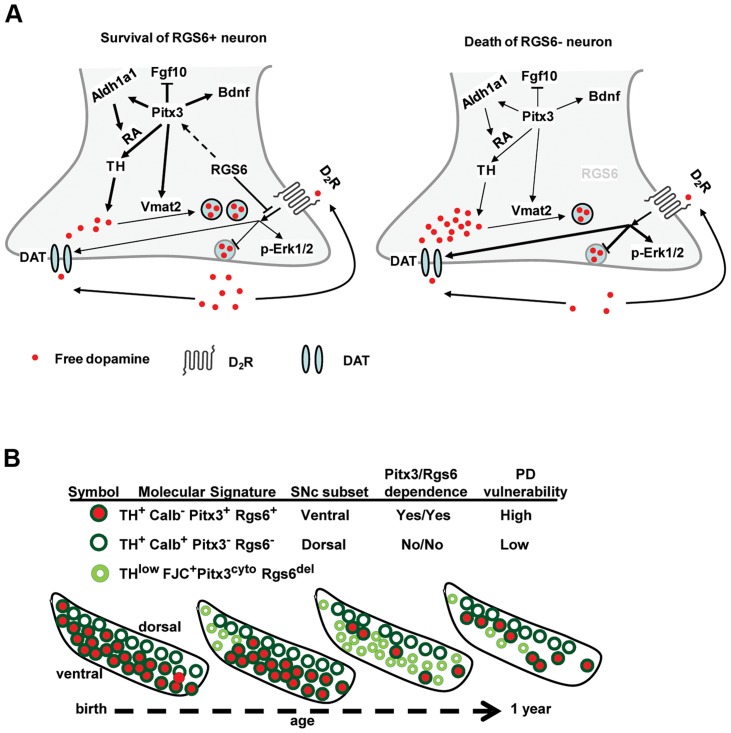
*Rgs6−/−* mice as a model for Parkinsonian neurodegeneration. (A) Rgs6-dependent signaling pathways in vSNc mDA neurons control Pitx3 expression, which itself controls expression of Aldh1a1, TH, Fgf10, Vmat2 and Bdnf. Together, Rgs6 and Pitx3 define a survival pathway in these neurons. The dopamine receptor Drd2 may be a target of Rgs6 action and consistent with this hypothesis, expression of the dopamine transporter DAT is up-regulated in *Rgs6^−/−^* vSNc. (B) Schematic representation of major subsets of SNc mDA neurons present at different ages in midbrains of *Rgs6* ^−/−^ mice showing progressive degeneration (small pale green) followed by loss of vSNc mDA neurons.

The regulatory action of Rgs6 was associated with various GPCRs, in particular the dopamine receptor D2 (Drd2) [25]. Ventral SNc mDA neurons are subject to regulatory negative feedback mediated by Drd2 autoreceptors. Expression of Drd2 itself is not affected in vSNc mDA neurons of *Rgs6* ^−/−^ mice (Fig. 8A) despite its dependence on Pitx3 [19]. Dopamine signalling in these neurons leads to activation of Erk1/2 [41] and accordingly, we observed significant phospho-Erk1/2 only in TH^low^ neurons of *Rgs6^−^* ^/−^ vSNc (Fig. 8B). In addition, enhanced DA signalling downstream of Drd2 [42] would be expected to increase expression of the dopamine transporter DAT (SLC6A3) which is otherwise dependent on Pitx3. It was indeed observed that activated glycosyl-DAT is high in TH^low^ neurons of *Rgs6* ^−/−^ vSNC (Fig. 8C). Thus, high DAT would presumably increase intracellular DA levels in these neurons by promoting DA uptake [43]. Cytoplasmic DA would be further enhanced by the decrease of Pitx3-dependent [43] Vmat2 (Fig. 7B) which is responsible for sequestration of DA into vesicles. Thus, the combined elevation of DAT with decreased Vmat2 is very likely to maintain high levels of free DA that may be toxic [44] and contribute to the degenerative process and cell death [45]. Increased DA signalling thus constitutes a putative mechanism to explain the late-onset neurodegeneration observed in *Rgs6* ^−/−^ vSNc mDA neurons (Fig. 9A).

The two *Rgs6^−/−^* phenotypes appear to reveal incremental penetrance of similar defects: in this case, it would be expected that the contralateral midbrain of affected mice will be affected at some point. We thus further scrutinized the affected *Rgs6^−/−^* midbrains with the clear unilateral deficits described above and found two that exhibited on the contralateral side, small clusters of mDA neurons of relatively normal appearance but that are TH^low^, have cytoplasmic Pitx3, high glycosylated DAT and enhanced phospho-Erk1/2 expression (Figure S4). Such cells were not observed in control midbrains. These data suggest that elevation of DA signalling subsequent to the loss of Rgs6 is closely associated with translocation of Pitx3 to the cytoplasm.

In summary, the loss of *Rgs6* is associated with three phenotypes each individually associated with mDA neuron degeneration, cell loss and Parkison's disease namely, 1) the loss of Pitx3 expression and of its target genes, 2) the loss of DJ-1 and finally 3) excessive dopaminergic tone.

## Discussion

A few gene expression profiling studies have compared gene expression in SNc versus VTA [46]–[48]. These studies identified large numbers of SN or VTA restricted genes but did not include criteria to relate these specificities to function. Our reliance on *Pitx3* gene dependence to identify genes with preferential expression in Pitx3-independent dSNC versus Pitx3-dependent vSNc neurons allowed the prioritization of candidate genes based on the pro-survival *Pitx3* gene. This approach also allowed definition of the unique expression profiles for dorsal compared to ventral SNc mDA neurons. We focused on vSNc-enriched and Pitx3-dependent genes in order to identify candidates for role(s) in vSNc mDA neurons survival. The list of 10 *Pitx3*-activated and 6 *Pitx3*-repressed genes in this subset includes only one known gene encoding a regulator of a signaling pathway, Rgs6. Further, Rgs6 is the most dependent on *Pitx3* for expression in VTA. The list also includes a transcriptional co-regulator Lmo3 that may contribute to Pitx3-dependent survival pathways but it's putative involvement in survival appeared less likely than Rgs6 to contribute to an age-dependent phenotype.

Thus, the present study focused on characterization of neuronal loss and on molecular features of degenerating vSNc neurons as a result of *Rgs6* inactivation. We observed late-onset degeneration of vSNc mDA neurons in *Rgs6* ^−/−^ midbrains (Fig. 9B). This phenotype is detected at about 6 months of age and becomes more important in 1y-old mice. At that age, clear mDA cell loss is observed in a subset of mice (Table S2). It is likely that the degenerating TH^low^ mDA phenotype (Fig. 5) and the mDA cell loss (Fig. 4) represent progressive steps of the same defects; accordingly, TH^low^ neurons are also observed in the vSNc of midbrains with cell loss.

This late-onset degeneration is similar to another mouse model of monogenic PD, the *DJ-1* (*Parkin 7*) mutant, that also initially exhibits unilateral defects [7]. Indeed, *DJ-1^−/−^* mice present unilateral loss of mDA cell bodies as early as 2 months after birth, with a transition to bilateral cell loss occurring at 1 year of age. Our study of *Rgs6* ^−/−^ mice showed unilateral cell loss, as evidence by decreased number of TH+ and NissL+ neurons (Fig. 4A–C, Table S2) at 12 months after birth, while evidence of TH^low^ degenerating neurons is readily apparent earlier at 6 months of age (Fig. 5A, Table S2). The comparison of phenotypes for *DJ-1* and *Rgs6* knockout mice indicates that: 1) they both have selective degeneration of SNc, but not VTA, mDA neurons, 2) they both have progressive degeneration and loss of mDA neurons, 3) degeneration begins unilaterally, 4) degeneration eventually becomes bilateral with earlier transition in *DJ-1^−/−^* than *Rgs6^−/−^* mice. A distinguishing feature of the *Rgs6^−/−^* model is the bias towards degeneration of Calb-negative vSNc mDA neurons compared to Calb-positive dSNc mDA neurons that remain largely unaffected (Fig. 5A, Figure S4), as is usually observed in PD.

The slow degeneration of TH^low^ vSNc neurons provided an opportunity to define the molecular features that accompany the dysmorphology. These neurons display increased expression of markers previously associated with pathological changes such as FluroJade C, LC3B and phospho-p27^Kip1^. Moreover, degenerating TH^low^ vSNc neurons show decreased DJ-1 and elevated Pink1 and Lrrk2 protein expression, suggesting a relationship between Rgs6 signaling and pathways implicated in PD pathology (Fig. 6). Future studies should address the relationships between DJ-1, Pink1 and Lrrk2 in degeneration pathways of *Rgs6^−/−^* mice in terms of their known roles in mitochondria dynamics, calcium, balance, redox state and cell signaling.

Collectively, the data show that Rgs6 signaling is necessary for maintenance of vSNc mDA neurons in the aging animal and that its downstream action may be mediated, at least in part, by Pitx3-dependent mechanisms (Fig. 9A). Indeed, the TH^low^ vSNc neurons exhibit low levels of Pitx3 and of its target gene products TH, Aldh1a1, Bdnf, Vmat2, together with enhanced expression of Pitx3-repressed Fgf10 (Fig. 7). The TH^low^ neurons also exhibit cytoplasmic Pitx3 staining suggesting that there may be regulation of nuclear-cytoplasmic localization: this effect could be mediated through phosphorylation of Pitx3 as it was suggested that phosphorylated Pitx1 has greater affinity for nuclear DNA binding than its de-phosphorylated form [49]. One of the documented Pitx3-activated factors, Bdnf, is an important mediator of the neuroprotective action of Pitx3 during development and could contribute to trophic impairment in adult degenerating neurons. The degenerating neurons also exhibit a loss of determinants of postmitotic dopaminergic identity (TH, Aldh1a1, Vmat2) and this likely affects their neuronal activity. Aberrant neuronal activity is a hallmark [45] of the pre-symptomatic stage of PD and this likely transitions to major cellular disruptions (proteosome dysfunction, mitochondrial integrity, calcium permeability…) associated with degeneration and cell death.

What could be the target of Rgs6 action? One likely possibility is the dopamine receptor D2 (Drd2). Indeed, Rgs6 is a negative modulator of GPCR activity, including the dopamine Drd2 receptor [25]. Drd2 expression itself was not affected in degenerating *Rgs6* ^−/−^ mDA neurons (Fig. 8A). However, the vSNc TH^low^ neurons showed evidence of increased DA signaling, namely accumulation of phospho-Erk1/2 (Fig. 8B) and enhanced glycosylated dopamine transporter (Slc6a3/DAT) expression in the mutant (Fig. 8C), consistent with a putative loss of negative Rgs6 input on dopamine signaling [50]. Since the *Pitx3^−/−^* midbrain exhibits decreased DAT and Drd2 [19], the observed increase in DAT together with phospho-Erk1/2 are consistent with a primary action of Rgs6 inactivation on DA signaling. Rgs6 may thus contribute to the auto-regulatory negative feedback of the dopaminergic system and its absence may lead to dopamine-dependent oxidative stress and neuronal loss [2], [50]. The enhanced phospho-p27^Kip1^ and FluoroJade staining support the interpretation that these cells are under stress.

Alternatively, Rgs6 may have GPCR-independent actions: those could involve the GDNF pathway that is essential for catecholaminergic neuron survival [24] or involve direct action on apoptotic pathways [39].

An important aspect of the expression changes discussed above is that they only occur in TH^low^ dysmorphic neurons that normally express Rgs6 and not in other mDA neurons of VTA and dSNc that are negative for Rgs6 (Fig. 3). Therefore, the concordance between Rgs6 midbrain expression and observed cell degeneration patterns suggests that the changes are cell-autonomous and directly related to Rgs6-dependent signaling operating in Rgs6+Pitx3+ vSNc neurons. We cannot however rule out the contribution of other brain systems affected by Rgs6 deficiency.

Collectively, the data show that Rgs6 signaling is necessary for maintenance of vSNc mDA neurons in the aging animal and that its downstream action may be mediated, at least in part, by Pitx3-dependent mechanisms (Fig. 9). The present work identified a critical signalling pathway that controls survival of the mDA neuron subset that preferentially degenerates in PD. Further dissection of this pathway may lead to therapeutically useful insights on the unique properties of this group of mDA neurons.

## Materials and Methods

### Ethics statement

All experimental procedures with laboratory animals were approved by the IRCM Animal Protection Committee and followed guidelines and regulations of the Canadian Council of Animal Care.

### Animal models

All mice were maintained as heterozygous carriers in the C57Bl/6J background and maintained on a 12 h light-dark cycle with food and water ad libitum. *Rgs6*-null [26] and *TH-EGFP* [51] mice were described previously. *Pitx3*-null mice were generated in this laboratory [52].

### Dissections and flow cytometry

Ventral midbrain dissections were performed on *WT* and *Pitx3* ^−/−^ newborn mice (P1–P4) crossed onto *TH-EGFP* heterozygous background. Mouse brains were quickly washed in ice-cold PBS and then placed into cold Hibernate-A/1%B27 solution (Gibco) to dissect EGFP+ ventral midbrain (vMB) tissue under the fluorescence stereoscope (Leica DFC300 FX). Tissue blocks of vMB were then further microdissected so as to separate SNc (lateral) from VTA (medial). VTA and SNc tissue blocks were digested using the papain dissociation kit (Worthington). Dissociated cells were then resuspended in warm Hibernate-A/1%B27 solution containing propidium iodide (PI, 1 µg/ml), passed through 100 µm mesh and sorted by flow cytometry using the MOFLO™ instrument (Beckman Coulter). PI−/EGFP+ live sorted cells were deposited in 30 µl of RNAlater solution (Ambion) (max. of 3000 cells per 30 µl of RNAlater) to preserve RNA integrity.

### RNA extraction, quantitative real-time PCR (qRT-PCR) and microarray analyses

Sorted EGFP^+^ cells in RNAlater were processed in batches of approximately 5000 cells for purification of total RNA using RNeasy Micro kit (Qiagen). Briefly, 350 µl of RLT lysis buffer was added per 30 µl RNAlater-suspended cells. After vortexing for 1 min, 1 volume of 70% ethanol was added and the content loaded into single pre-equilibrated RNeasy MinElute column. This was done in duplicate for each of the four different preparations of EGFP^+^ cells (Pitx3^+/+^ SNc, Pitx3^+/+^ VTA, Pitx3^−/−^ SNc, Pitx3^−/−^ VTA). Column-bound RNA was washed as recommended and eluted with 14 µl of RNAse-free water. Quality of total RNA was verified with the Agilent RNA 6000 Nano kit adapted for Agilent 2100 Bioanalyzer.

For RT-qPCR, first-strand cDNA was synthesized using Superscript III RT enzyme and accompanying kit (Invitrogen). Primers for PCR amplification are displayed in Table S3. qPCR was performed using Perfecta reagents (Quanta) on a MX-3005 device (Stratagene), and results were analyzed using the accompanying software. All quantifications were relative to Gapdh mRNA.

For microarray hybridization, total RNA with minimal degradation was used. Prior to hybridization onto Affymetrix Gene1.0ST expression arrays, which was done at the Genome Quebec/McGill Innovation Centre, total RNA was linearly amplified using WT-Ovation Pico RNA Amplification kit (NuGen). Gene expression summary values were computed by RMA Express (Bolstad et al, 2003) and raw data was normalized with the LPE algorithm embedded in the FlexArray suite of programs (Genome Quebec). Differentially expressed genes were chosen on the basis of their p-value≥0.05, fold-enrichment ≥1.5 and raw array signal ≥60. Hierarchical clustering and heat map display was done using Genesis (Institute for Genomics and Bioinformatics, Austria)

### Immunohistochemistry

Mice were anesthetized and perfused intra-cardially with fresh 4% paraformaldehyde/PBS buffer. Brains were collected and post-fixed for 24 h at 4°C. After inclusion in paraffin, brains were cut in 5–6 µm coronal sections using microtome and mounted on Superfrost Plus (Fisher Scientific) slides. Immunonohistochemistry was performed after paraffin removal and hydration through xylene and graded alcohol series. Antigen retrieval was performed in 10 mM sodium citrate (pH 8.5) at 80°C water bath for 30 min. Sections in citrate solution were left to cool to room-temperature (RT) after which a step of endogenous biotin block was performed (Streptavidin/biotin kit, Vector Labs). Blocking with 5% normal serum for 1 h at RT preceded primary antibody incubation (overnight at 4°C). Primary antibodies used were against Pitx3 (rabbit home-made, 1∶400), Rgs6 (rabbit home-made 1∶25), Th (Millipore MAB318, 1∶1000), Th (Millipore AB152, 1∶500), Calb1 (R&D Systems AF3320, 1∶40), Fgf10 (Millipore ABN44, 1∶1000), Slc6a3 (Santa Cruz sc-32258, 1∶250), Drd2 (Santa Cruz sc5303, 1∶100), Aldh1a1 (Abcam ab24343, 1∶400), BDNF (Abcam ab108319, 1∶25), phospho-p27 (Abcam ab32096, 1∶50), phospho-Erk1/2 (Cell Signaling 4376, 1∶25), DJ-1 (Abcam ab18257, 1∶500) Lrrk2 (Epitomics 3514-1, 1∶50), Pink1 (Novus Biologicals BC100-494, 1∶50), LC3B (Cell Signaling 3868, 1∶50), and Vmat2 (Millipore AB1598P, 1∶200). Secondary antibodies were either biotinylated (Vector Labs, 1∶250) or directly coupled to fluorochromes such as AlexaFluor 488/546 (Invitrogen, 1∶250). For ABC method of amplification, AlexaFluor350/480/546- or HRP-conjugated (PerkinElmer NEL750, 1∶1000) streptavidin compounds were used. For immunoperoxidase staining, 1% hydrogen peroxide treatment was done just after antigen retrieval step. Mounting of sections was in Mowiol (Sigma) (fluorescence) or in Permount (Fisher Scientific) (DAB chromogen reaction). Immunofluorescence sections were observed on Leica DM6000B light microscope and Carl Zeiss LSM700 confocal microscope.

### Fluoro-Jade (FJC) and Nissl staining

FJC (Molecular Probes, 0.01% stock in water) staining was performed just after the immunohistochemical procedure [33], [34]. Briefly, sections were washed in water, dipped for 5 min in 0.06% of potassium permanganate, rinsed again in water and placed in 0.0001% FJC/0.1% acetic/0.0001% DAPI solution for 10 min. Finally, slides were rinsed in water and mounted in 0.1% acetic acid/80% glycerol (v/v). The Fluorescein/FITC filter system was used to visualize FJC. Nissl staining was performed on deparaffinized sections by immersion into warm 0.1% cresyl violet solution for 10 min, rinsing three times in distilled water and differentiating in 95% ethanol. Slides were then dehydrated in 100% alcohol, cleared in xylene and mounted with Permount.

### Cell counts and statistics

Cell counts were performed using ImageJ (National Institutes of Health). For cell counts of degenerating neurons, TH-stained or TH/FJC/Dapi-stained coronal sections were loaded on ImageJ; the sections spanned regular intervals (30 or 100 µm) across rostro-caudal extent of midbrain of *WT* (n = 2) and 1-yo *Rgs6* ^−/−^ mice (n = 2). For each section, total numbers of TH+, TH+/FJC+ and TH−/FJC+ cells were separately counted for SNc and VTA in both hemispheres. The percentage of TH^+^ degenerating neurons for each anatomical region reflects the ratio between the total number of TH+/FJC+ events and the total number of TH+ events for all rostro-caudal series. Values are reported as means +/−S.D. Statistical significance was calculated using Student T-test.

## Supporting Information

Figure S1 Immunoperoxidase staining for TH on coronal sections of 1 y-old WT and *Rgs6^−/−^* midbrains across the rostrocaudal extent (Bregma reference values shown on left) of the midbrain. Scale bar 100 µm.
(TIF)

Figure S2 Immunoperoxidase staining for TH on coronal sections of 1 y-old WT (A–D) and *Rgs6^−/−^* (E–J) midbrains at comparable rostrocaudal levels (Bregma −3.16 mm). TH+ neurons in SNc and VTA are shown in magnification to emphasize differences and similarities in the appearance of mDA neurons, including their size, morphology and fiber networks between genotypes. Scale bar 200 µm.
(TIF)

Figure S3 Normal dorsal SNc Calb1+ mDA neurons in aging *Rgs6* ^−/−^ mice. Immunohisto-fluorescence staining for TH (green) and Calb1 (red) in coronal sections of 1 y-old *Rgs6* ^−/−^ and *WT* midbrains. mDA neurons of vSNc do not express Calb1 unlike those of dSNc. In *Rgs6* ^−/−^ mice, Calb1− vSNc mDA neurons are dysmorphic (TH^low^), but Calb+ dSNc neurons appear normal (TH^high^). Scale bar 50 µm.
(TIF)

Figure S4 Clusters of abnormal mDA vSNc neurons on contralateral side of *Rgs6^−/−^* midbrains that have vSNc deficits on the other side. Double immunofluorescence staining for TH (green) and (*A*) DAT (red), (*B*) phospho-Erk1/2 (red) and (*C*) Pitx3 (red) on coronal sections of 1 y-old *Rgs6^−/−^* mice showing mDA clusters of abnormal cells, ie with increased DAT (A), phospho-Erk1/2 (B) and cytoplasmic Pitx3 (C). Arrowheads indicate unaffected neurons and arrows point to affected vSNc neurons. Scale bar 20 µm.
(TIF)

Table S1 Lists of mDA subset-specific probesets (genes) corresponding to the clusters presented in Figure 2. The expression ratios are shown in base 2 log for each probeset.
(XLSX)

Table S2 Summary of midbrain dopaminergic defects in *Rgs6* ^−/−^ and *Pitx3* ^−/−^ mice. *WT*, *Rgs6* ^−/−^ and *Pitx3* ^−/−^ mice of different ages (in days) were examined for defects in mDA neurons of SNc and VTA. Plus signs represent the numbers of mDA neurons and the estimated degree to which they are affected. Last column indicates the ratio of mice that displayed a phenotype compared to total.
(DOC)

Table S3 Primers used for qRT-PCR.
(DOC)
