# Supplementary figures and images for: Rgs6 is Required for Adult Maintenance of Dopaminergic Neurons in the Ventral Substantia Nigra
Source: PLoS Genet. 2014 Dec 11;10(12):e1004863. doi: 10.1371/journal.pgen.1004863 (PMC4263397; doi:10.1371/journal.pgen.1004863)

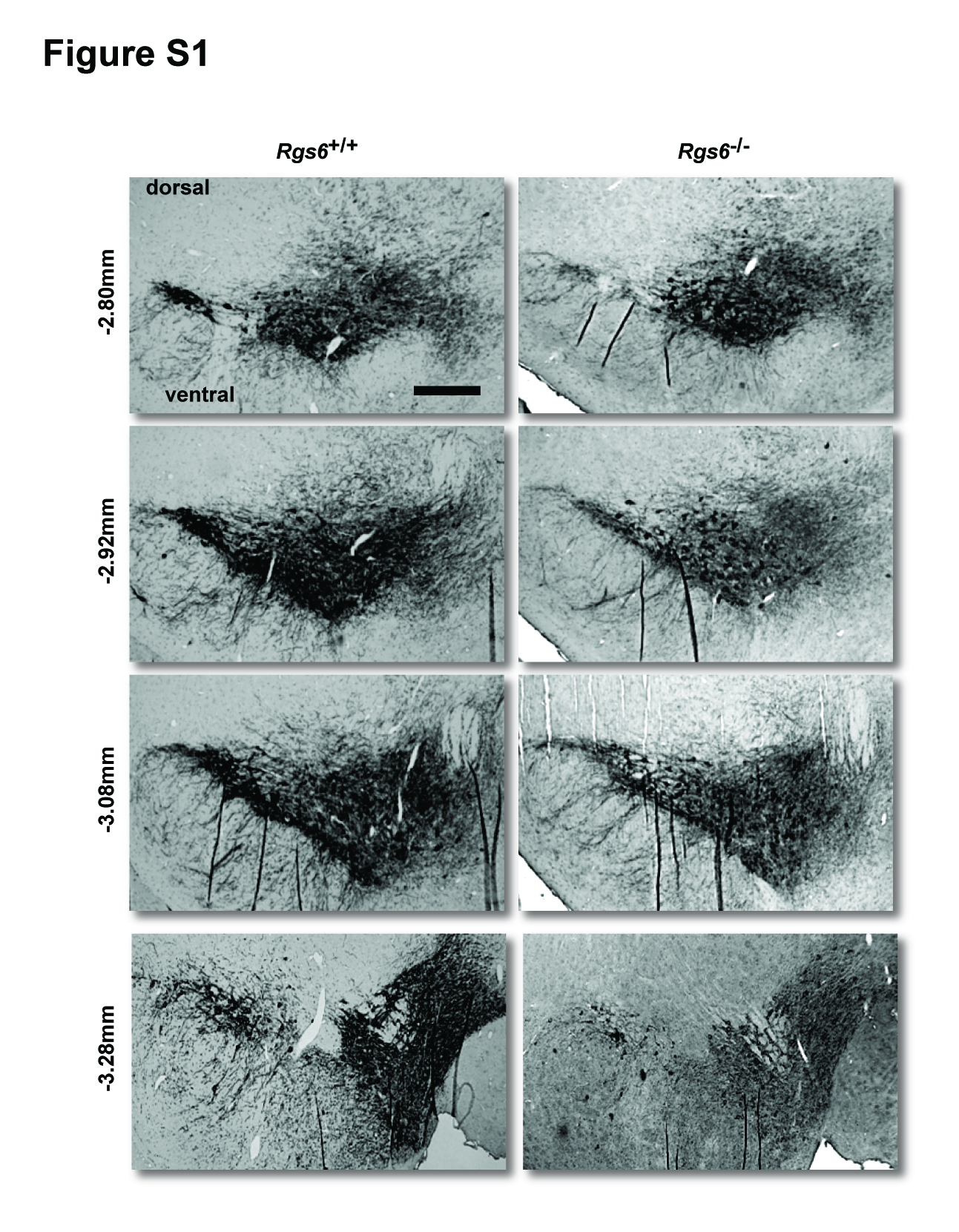

Supplement: Figure S1 — Immunoperoxidase staining for TH on coronal sections of 1 y-old WT and Rgs6−/− midbrains across the rostrocaudal extent (Bregma reference values shown on left) of the midbrain. Scale bar 100 µm. (TIF) [file pgen.1004863.s001.tif]

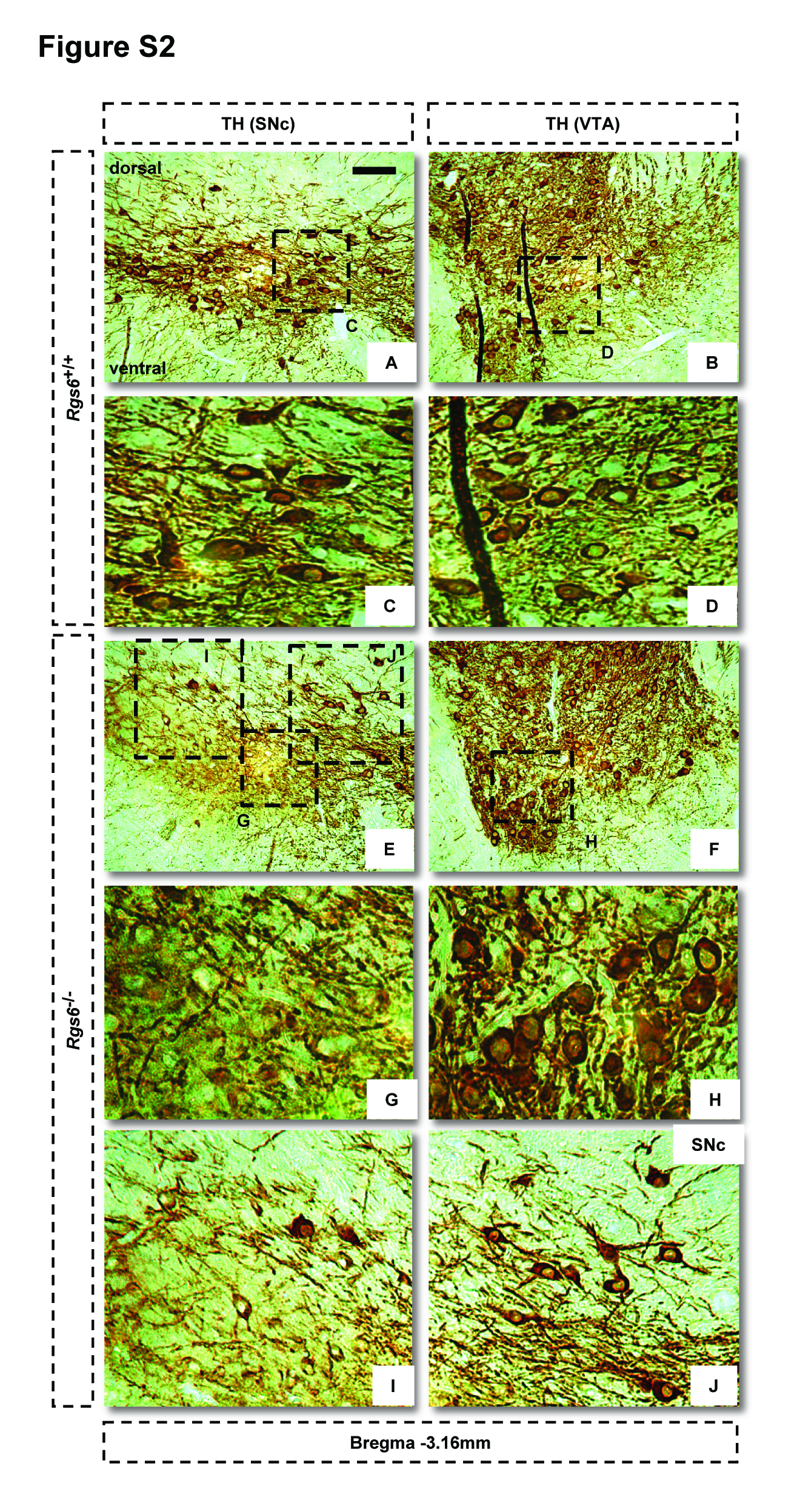

Supplement: Figure S2 — Immunoperoxidase staining for TH on coronal sections of 1 y-old WT (A–D) and Rgs6−/− (E–J) midbrains at comparable rostrocaudal levels (Bregma −3.16 mm). TH+ neurons in SNc and VTA are shown in magnification to emphasize differences and similarities in the appearance of mDA neurons, including their size, morphology and fiber networks between genotypes. Scale bar 200 µm. (TIF) [file pgen.1004863.s002.tif]

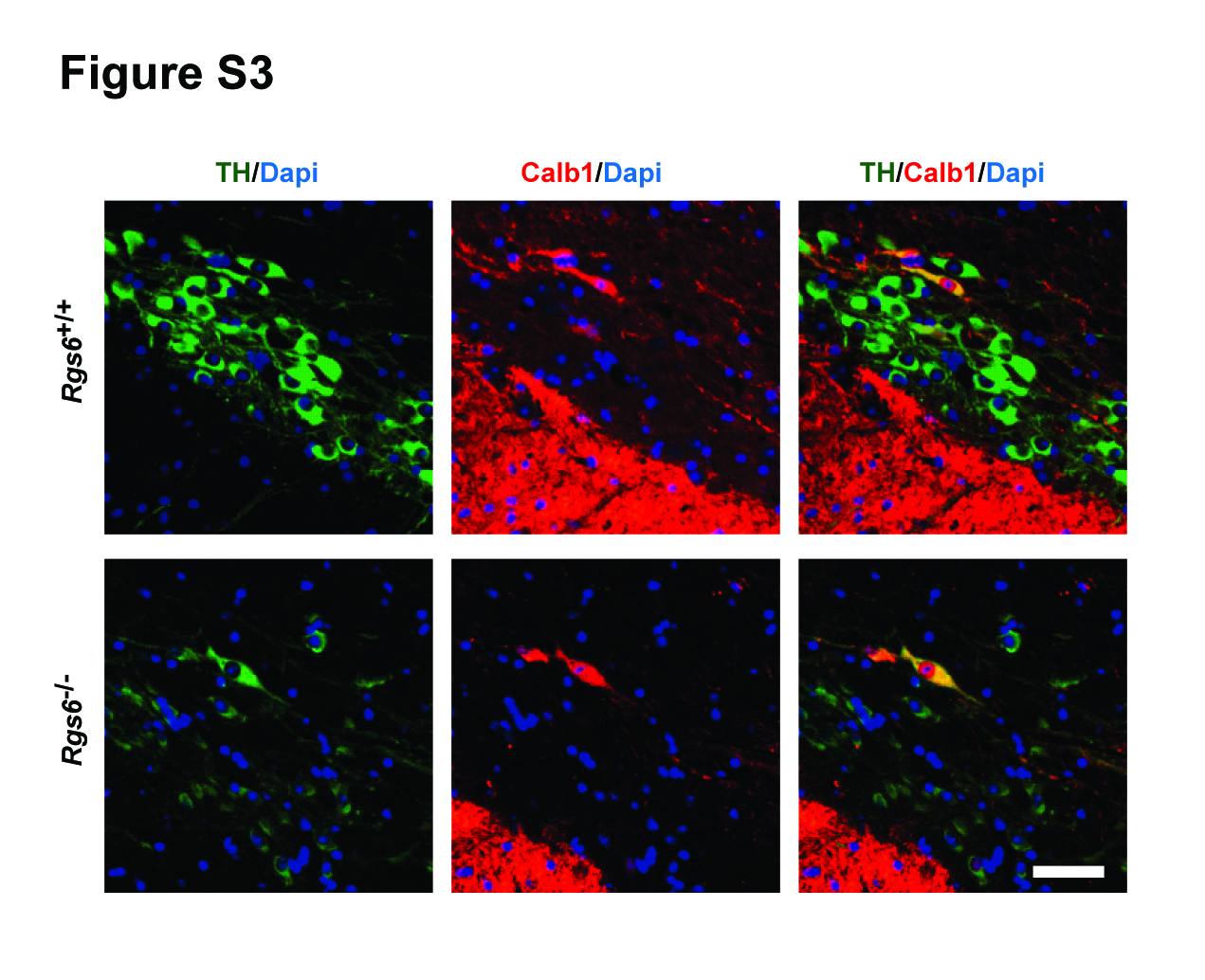

Supplement: Figure S3 — Normal dorsal SNc Calb1+ mDA neurons in aging Rgs6 −/− mice. Immunohisto-fluorescence staining for TH (green) and Calb1 (red) in coronal sections of 1 y-old Rgs6 −/− and WT midbrains. mDA neurons of vSNc do not express Calb1 unlike those of dSNc. In Rgs6 −/− mice, Calb1− vSNc mDA neurons are dysmorphic (THlow), but Calb+ dSNc neurons appear normal (THhigh). Scale bar 50 µm. (TIF) [file pgen.1004863.s003.tif]

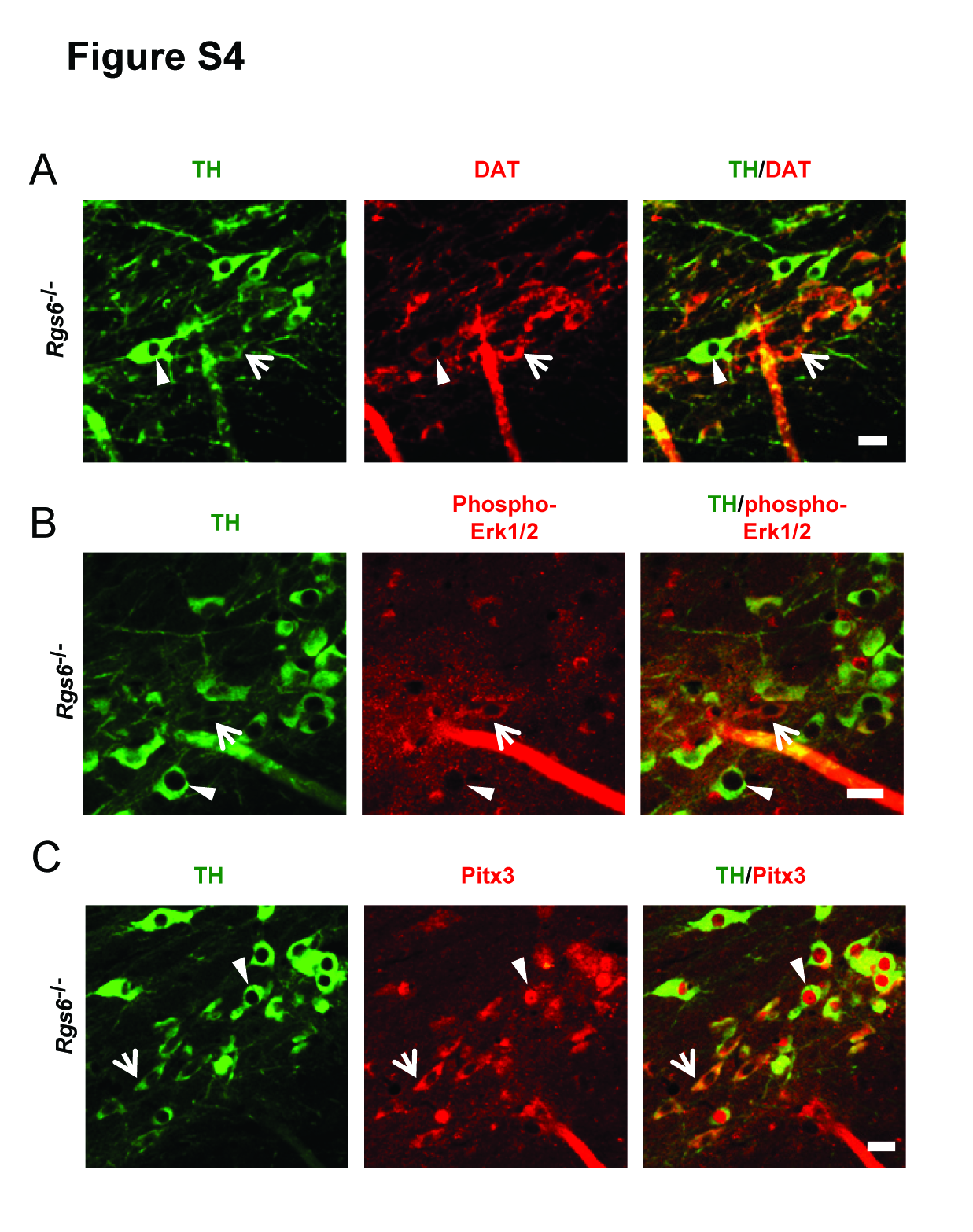

Supplement: Figure S4 — Clusters of abnormal mDA vSNc neurons on contralateral side of Rgs6−/− midbrains that have vSNc deficits on the other side. Double immunofluorescence staining for TH (green) and (A) DAT (red), (B) phospho-Erk1/2 (red) and (C) Pitx3 (red) on coronal sections of 1 y-old Rgs6−/− mice showing mDA clusters of abnormal cells, ie with increased DAT (A), phospho-Erk1/2 (B) and cytoplasmic Pitx3 (C). Arrowheads indicate unaffected neurons and arrows point to affected vSNc neurons. Scale bar 20 µm. (TIF) [file pgen.1004863.s004.tif]
