# Supplementary material for: Rgs6 is Required for Adult Maintenance of Dopaminergic Neurons in the Ventral Substantia Nigra
Source: PLoS Genet. 2014 Dec 11;10(12):e1004863. doi: 10.1371/journal.pgen.1004863 (PMC4263397; doi:10.1371/journal.pgen.1004863)
Supplement: Table S2 — Summary of midbrain dopaminergic defects in Rgs6 −/− and Pitx3 −/− mice. WT, Rgs6 −/− and Pitx3 −/− mice of different ages (in days) were examined for defects in mDA neurons of SNc and VTA. Plus signs represent the numbers of mDA neurons and the estimated degree to which they are affected. Last column indicates the ratio of mice that displayed a phenotype compared to total. (DOC) [file pgen.1004863.s006.doc]

**Table S2**

| **Genotype** | **Age (days)** | **TH signal** **(VTA)** | **TH signal (SNc)** | **Number of mice** |
| --- | --- | --- | --- | --- |
| ***WT*** | 0, 180, 365 | ++++ | ++++ | 32/32 |
| ***Rgs6*-/-** | 6 | ++++ | ++++ | 4/4 |
| ***Rgs6*-/-** | 180 | ++++ | +++ | 2/5  (partial degeneration) |
| ***Rgs6*-/-** | 365 | ++++ | ++ | 2/7 (cell loss)  3/7 (degeneration) |
| ***Pitx3*-/-** | 6 | ++++ | + | 4/4 |
| ***Pitx3*-/-** | 150 | ++ | + | 4/4 |
| ***Pitx3*-/-** | 365 | ++ | + | 4/4 |
