# Supplementary material for: Rgs6 is Required for Adult Maintenance of Dopaminergic Neurons in the Ventral Substantia Nigra
Source: PLoS Genet. 2014 Dec 11;10(12):e1004863. doi: 10.1371/journal.pgen.1004863 (PMC4263397; doi:10.1371/journal.pgen.1004863)
Supplement: Table S3 — Primers used for qRT-PCR. (DOC) [file pgen.1004863.s007.doc]

| **Gene** | **Primer Sequence (sens)** | **Primer Sequence (antisens)** |
| --- | --- | --- |
| **Aldh1a1** | TGTCATCTGCTCTGCAGGCTGG | TTGAATCCACCGAAGGGGCA |
| **Calb1** | TGACGGAAAGCTGGAACTGACAGA | CACAGATCTTTCAGCAAAGCATCCAGC |
| **Gapdh** | TGCAGTGGCAAAGTGGAGAT | ACTGTGCCGTTGAATTTGCC |
| **Kcnj6/Girk2** | AGATTGTGGTCATCCTGGAGGGAA | TTCCAGCGTTAGGACAGGTGTGAA |
| **Otx2** | ATGAGGGAAGAGGTGGCACTGAAA | TGTTCTGACCTCCATTCTGCTGCT |
| **Rgs6** | AATCTCAGGTTCTGGCTGTCTGTC | TTGCCAGATTTCCTCCACCCTCTT |
| **Slc6a3/DAT** | GGCCTATGCCATCACACCTGAG | TGTCCCCGCTGTTGTGAGATG |

**Table S3**
